# Optimised Extraction and Preliminary Characterisation of Mannoproteins from Non-*Saccharomyces* Wine Yeasts

**DOI:** 10.3390/foods10050924

**Published:** 2021-04-22

**Authors:** Carla Snyman, Julie Mekoue Nguela, Nathalie Sieczkowski, Matteo Marangon, Benoit Divol

**Affiliations:** 1 South African Grape and Wine Research Institute, Department of Viticulture and Oenology, Private Bag X1, 7602 Matieland, South Africa; carlasnyman@sun.ac.za; 2 Department of Agronomy, Food, Natural Resources, Animals and Environment (DAFNAE), University of Padova, Viale Dell’Università 16, Legnaro, 35020 Padova, Italy; matteo.marangon@unipd.it; 3 Lallemand SAS, 19 Rue des Briquetiers, BP 59, 31702 Blagnac, France; jmekoue@lallemand.com (J.M.N.); nsieczkowski@lallemand.com (N.S.)

**Keywords:** mannoprotein, yeast, non-*Saccharomyces*, extraction, wine, ultrasound, β-glucanase

## Abstract

The exogenous application of yeast-derived mannoproteins presents many opportunities for the improvement of wine technological and oenological properties. Their isolation from the cell wall of *Saccharomyces* *cerevisiae* has been well studied. However, investigations into the efficiency of extraction methods from non-*Saccharomyces* yeasts are necessary to explore the heterogeneity in structure and composition that varies between yeast species, which may influence wine properties such as clarity and mouthfeel. In this study, nine yeast strains were screened for cell wall mannoprotein content using fluorescence microscopy techniques. Four species were subsequently exposed to a combination of mechanical and enzymatic extraction methods to optimize mannoprotein yield. Yeast cells subjected to 4 min of ultrasound treatment applied at 80% of the maximum possible amplitude with a 50% duty cycle, followed by an enzymatic treatment of 4000 U lyticase per g dry cells weight, showed the highest mannoprotein-rich yield from all species. Furthermore, preliminary evaluation of the obtained extracts revealed differences in carbohydrate/protein ratios between species and with increased enzyme incubation time. The results obtained in this study form an important step towards further characterization of extraction treatment impact and yeast species effect on the isolated mannoproteins, and their subsequent influence on wine properties.

## 1. Introduction

The application of bioactive molecules for the improvement of industrial processes and products such as winemaking and wine is a topic of great interest, especially when these compounds originate from indigenous sources. Yeast-derived mannoproteins represent one such category of valuable compounds. They constitute a group of glycoproteins which, along with β-linked glucans and chitin, form the major components of the yeast cell wall [1]. In the well-studied genus *Saccharomyces*, the glycan moiety of mannoproteins consists mostly of mannose (>90%) and glucose, as well as N-acetyl-glucosamine and mannosylphosphate (0.1–1%), with a protein content ranging between 1% and 10% [2,3]. Their molecular weight may vary between 5 and 800 kDa, although the typical reported range is 50–500 kDa [3].

The molecular and structural properties of mannoproteins make them attractive to any food and beverage industry that may benefit from their many techno-functional properties [4]. Due to their amphiphilic nature, for example, mannoproteins show promise as stabilizers in food emulsions such as mayonnaise and salad dressing [5]. Furthermore, the application potential of mannoproteins for the improvement of wine technological and organoleptic properties is vast. During the alcoholic fermentation of wine and wine aging, mannoproteins are released by the fermenting yeast *Saccharomyces cerevisiae* into the wine matrix where they have been shown to positively affect various quality parameters such as protein and tartrate stability, mouthfeel, astringency, color and foaming in sparkling wines [6,7,8,9,10,11,12,13,14]. These desirable attributes have led to the adoption of winemaking practices such as the inoculation of high mannoprotein-producing yeast strains and aging on the lees for the promotion of yeast autolysis and enhanced mannoprotein release [15,16]. Furthermore, it has recently been proposed that, although traditionally considered as waste by-products, yeast lees originating from wine fermentation can also be collected and recycled within the wine industry for their above-listed properties [17].

However, there are several drawbacks associated with the reliance on fermenting or autolyzing cultures for the release of mannoproteins into the wine matrix. Besides the microbiological and organoleptic risks involved, aging on the lees is a time- and resource-consuming practice [18]. Additionally, the levels of mannoprotein released are frequently too low to be of commercial significance [19]. Furthermore, mannoprotein structural and chemical composition is directly influenced by the yeast growth phase and environmental conditions, which vary throughout the winemaking process [2,20,21,22,23]. Compositional and structural changes have, in turn, shown a strong impact on their effectiveness in addressing wine quality parameters such as protein haze formation and the modulation of astringency, possibly through altering their behavior in relation to certain wine macromolecules involved in this phenomenon [19,24,25,26]. For example, changes in the mannose/glucose ratio of mannoproteins has been shown to influence tannin stability as well as protein aggregation, compounds which are involved in astringency and haze formation, respectively [24,26]. Additionally, the haze protective ability of glycoproteins in wine has frequently been associated with a relatively high proportion of carbohydrate to protein [19].

The isolation of mannoproteins from the yeast cell wall therefore provides the opportunity for in-depth structural characterization before its exogenous addition to must or wine, in order to elucidate the relationship between structural variations and their impact on wine properties. Indeed, the addition of exogenous mannoprotein-containing products derived from the cell wall of the wine yeast *S. cerevisiae* is a fairly common winemaking practice [18]. However, another important determinant of mannoprotein physical structure and a topic for consideration is that of yeast strain and species [27]. Aspects such as total charge, charge distribution and accessible surface area vary between strains due to changes in oligosaccharide composition and levels of phosphorylation, which, in turn, influence their adsorption properties and interaction with wine macromolecules involved in wine quality [27]. The influence of yeast species other than *S. cerevisiae* on the structural composition of mannoproteins, and the subsequent impact on wine properties, is therefore an interesting avenue of investigation. Non-*Saccharomyces* yeast strains, including *Schizosaccharomyces pombe*, *Pichia fermentans*, *Metschnikowia pulcherrima*, *Saccharomycodes ludwigii*, *Torulaspora delbrueckii*, *Lachancea thermotolerans* and *Wickerhamomyces anomalus*, have demonstrated the ability to produce and release mannoproteins into the wine matrix, the chemical compositions of which often show considerable variation [28,29]. For example, mannoproteins released by *T. delbrueckii* during aging on the lees improved mouthfeel properties and color stabilization, whereas sequential fermentations of *S. pombe* and *L. thermotolerans* improved the sensory and aromatic characteristics of wine, possibly due to the ability of the released mannoproteins to retain positive aroma compounds such as B-ionone [30,31]. The extraction of mannoproteins from non-*Saccharomyces* yeasts provides an attractive alternative to their use as starter cultures to wine fermentation, especially from those known for their weak fermentative abilities or contribution of spoilage characteristics. Such a strategy would avoid potential drawbacks such as stuck fermentations, decreased wine quality, or inadequate release of mannoproteins due to the sub-optimal winemaking conditions and competition with *S. cerevisiae* [32].

While the heterogeneity of mannoproteins makes them a potentially useful application to the food and beverage industry due to the range of benefits they may impart, it also poses a challenge to their isolation from the yeast cell wall. Mannoproteins may be non-covalently bound, bound covalently to the cell wall glucan, or disulfide bound to other proteins that are covalently bound to glucan [4]. Due to the various ways in which mannoproteins are bound, different treatments are necessary to release them [33]. Non-covalently bound mannoproteins loosely associated with the cell wall are frequently extracted through physical and chemical methods such as heat or Sodium Dodecyl Sulphate (SDS) treatment [4]. Mechanical disruption using ultrasound is another, non-degrading method that has shown promising results for the release of mannoproteins [34]. Covalently bound mannoproteins, on the other hand, are typically released either through various acidic and alkaline washes which is likely to cause degradation of, or alterations to, the obtained glycocompounds, or through enzymatic treatment using β-glucanases [4,35]. In fact, the use of different treatments has shown to influence the techno-functional properties of the obtained extract and its impact on wine quality parameters [6,17]. Therefore, a combination of physical and enzymatic extraction methods is likely to yield the most representative pool of mannoproteins contained by the yeast cell wall.

This study aimed to screen the in-situ levels of mannoprotein contained in the cell wall of eleven wine yeast species using fluorescence microscopy methods, and subsequently optimize the extraction of mannoproteins from four selected strains using various ultrasound and enzymatic treatments. The present goal was to extract a high amount of heterogeneous mannoproteins representative of those in the cell wall, and to perform a preliminary investigation into the compositional differences of the extracts obtained from the different species and by different treatments.

## 2. Materials and Methods

### 2.1. Yeast Strains and Cultivation

Wine strains SC01, SB62, MF77 and TD70 respectively from the species *Saccharomyces cerevisiae*, *Saccharomyces boulardii*, *Metschnikowia fructicola* and *Torulaspora delbrueckii* used in this study were obtained from Lallemand Inc (Montréal, QC, Canada). Additional yeast strains included in the initial cell wall screening using fluorescent microscopy techniques include *Saccharomyces paradoxus* R088 [36], *Pichia fermentans* IWBT Y1164, *Saccharomycodes ludwigii* IWBT Y0154 (South African Grape and Wine Research Institute, Stellenbosch University, Stellenbosch, South Africa), *Metschnikowia pulcherrima* MP74 (Lallemand Inc, Montréal, QC, Canada), and *Schizosaccharomyces pombe* YMV1550 (Department of Microbiology, Stellenbosch University). Cultures were maintained in 30% glycerol and cultivated at 30 °C on yeast peptone dextrose (YPD) agar (Biolab diagnostics, Wadenville, South Africa). Yeast pre-cultures were prepared by inoculating test tubes containing 10 mL YPD broth with a single yeast colony and allowing propagation at 30 °C on a test-tube rotor overnight. For fluorescence microscopy analysis and mannoprotein extractions, aliquots of pre-culture were inoculated into 200 mL enrichment media containing yeast extract (10 g/L), peptone (20 g/L) and glucose (20 g/L) to reach an optical density of 0.1 at 600 nm, prepared in 0.1 M McIlvaine’s buffer adjusted to pH 5, and cultured at 30 °C with shaking at 120 rpm, as described by Aguilar-Uscanga and François [37] for improved cell wall mannan content.

### 2.2. Fluorescence Microscopy Techniques

#### 2.2.1. Staining Procedure

The FITC-ConA (Concanavalin A from *Canavalia ensiformis* conjugated to the FITC fluorescent dye, Sigma-Aldrich, Modderfontein, South Africa) staining procedure applied in this study was adapted from Okada and Ohya [38]. Yeast samples were collected in duplicate at 0 h (upon inoculation), 9 h (exponential phase of growth), 48 h (early stationary phase of growth) and 7 days (late stationary phase of growth) after inoculation into the enrichment medium. Samples were centrifuged at 6000× *g* for 5 min, the supernatant was discarded, and cells were resuspended in 0.1 M phosphate buffer, pH 7.2. After the suspensions were again centrifuged and the supernatant discarded, cells were resuspended in phosphate buffer containing 20 µg/mL FITC-ConA to reach an optical density of 0.1 at 600 nm. Suspensions were incubated for 30 min at 30 °C, after which they were again centrifuged and the supernatant discarded, and resuspended in phosphate buffer. Aliquots of 20 µL from each sample were mounted in duplicate onto glass slides and covered with glass cover slips.

#### 2.2.2. Fluorescence Imaging

Cells were visualized with a Zeiss Axio Scope A1 light microscope attached to a Colibri 7 LED light source attachment with channels for fluorescence excitation. Five images were acquired per mounted slide excited by the blue excitation line (469/38 nm), under constant exposure time of 167.41 ms for all samples, using the Zeiss Axiocam 208 and Zeiss ZEN software. Subsequent image analysis was performed using ImageJ software (National Institutes of Health, Bethesda, MD, USA). The corrected total cell fluorescence (CTCF) was calculated for the cells captured in each image, for normalization of cell fluorescence intensity by background fluorescence (CTCF = integrated density of the cell − (cell area × mean fluorescence of 5 background readings)) [39], and was further normalized by cell area to account for differences in cell size.

### 2.3. Mannoprotein Extraction Optimisation

#### 2.3.1. High-Intensity Ultrasound Treatment

Yeast cells were collected after 48 h growth in enrichment medium, centrifuged, and the supernatant discarded. Cells were subsequently resuspended in 0.1 M phosphate buffer, pH 6.5, at a concentration of 2.5 × 10^8^ cells/mL, and placed on ice for the duration of ultrasound treatment using a 20 kHz horn-type sonicator (Fisherbrand™ Model 120 Sonic Dismembrator, Fisher Scientific, Loughborough, UK) equipped with a 6 mm probe. Ultrasound treatment variables included duty cycle (20%, 50% and 80% of total treatment time in which sonication was occurring), the ultrasound amplitude (20%, 50% and 80% of the maximum amplitudes that can be delivered by the sonicator, which corresponds to the ultrasound intensities 7.01, 24.76 and 53.05 W/cm^2^, respectively), and total duration of ultrasound treatment (1, 2, 3, 4, 7 and 10 min). After sonication treatment, samples were centrifuged at 4500× *g* for 10 min, and the supernatant was collected for optical density measurements at 280 nm (NanoDrop^®^ ND-1000 spectrophotometer, NanoDrop Technologies LLC, Wilmington, DE, USA). The pellets from selected samples were subjected to subsequent enzymatic treatments.

#### 2.3.2. Enzymatic Treatment

Enzymatic treatment of yeast cells collected either after 48 h growth in enrichment medium or after ultrasound treatment was carried out using lyticase from *Arthrobacter luteus* (β-1,3-glucanase, Sigma-Aldrich). Cells were resuspended in phosphate buffer (pH 6.5), and lyticase was added to yield 250, 500, 1000, 2000 or 4000 units of enzyme per g dry weight of cells. The enzymatic treatment was carried out at 37 °C for 0.5, 1, 2, 4, 8 or 20 h, and thereafter inactivated at 60 °C for 10 min. Suspensions were subsequently centrifuged at 2000× *g* for 10 min, and the supernatant was collected for mannoprotein precipitation, and protein and carbohydrate quantification.

#### 2.3.3. Protein and Carbohydrate Quantification

The collected supernatant was added to 100% acetone (3:1, acetone:supernatant) and left at −20 °C overnight before centrifugation at 21,380× *g* for 30 min. The pellet was resuspended in Milli-Q^®^ water and total protein concentrations were determined using the Pierce^®^ BCA Protein Assay kit (Thermo Scientific) according to the manufacturer’s instructions. Total carbohydrate content was determined using the phenol sulphuric acid test, estimated from a standard curve constructed with mannose [40]. In a 96-well microplate, 150 µL sulphuric acid was added to 50 µL sample or mannose, to which 30 µL phenol (5% *w*/*v*) was subsequently added, and the plate was incubated at 30 °C for 20 min. Colorimetric detection of the protein and carbohydrate assays was performed by measuring absorbance at 540 nm and 490 nm, respectively, using a PowerWave™ Microplate Scanning Spectrophotometer (BioTek Instruments Inc, Winooski, VT, USA).

#### 2.3.4. Protein and Carbohydrate Visualization

Proteins and carbohydrates were visualized through the use of sodium dodecyl sulphate polyacrylamide gel electrophoresis (SDS-PAGE) as previously described [41]. Gels containing 15% bis-acrylamide were loaded with 40 µL of protein samples suspended in a loading buffer with final concentration of 0.0175 M Tris-HCl (pH 6.8), 0.8% SDS, 9% glycerol, 2.5% β-mercaptoethanol and 0.002% bromophenol blue, and run on a Bio-Rad Mini-Protean^®^ Tetra Cell System (Bio-Rad Laboratories, Hercules, CA, USA). Electrode chambers were filled with Tris-Glycine buffer (50 mM Tris, 200 mM glycine, 0.2% SDS). For protein visualization, gels were stained overnight in staining solution (1 g Coomassie blue R250 (Merck, Darmstadt, Germany) in 50% (*v*/*v*) ethanol, 10% (*v*/*v*) acetic acid), and destained with 12.5% isopropanol and 10% (*v*/*v*) acetic acid. For carbohydrate visualization, gels were stained using the periodic acid-Schiff (PAS) protocol described by Kapitany and Zebrowski [42]. Images of the gels were captured using a Molecular Imager^®^ Gel Doc^™^ System (Bio-Rad Laboratories) using Image Lab^™^ Software v6.0 (Bio-Rad Laboratories).

### 2.4. Statistical Analysis

Statistical analyses were performed using the computer software GraphPad Prism v. 8.0.2 (GraphPad, San Diego, CA, USA). The significant differences between treatments and time-points were determined by one-way and two-way analysis of variance (ANOVA) followed by Fisher’s LSD with statistical significance determined using an alpha value of 0.05.

## 3. Results

The data obtained in this study can be divided into two main sections, namely those of fluorescence microscopy screening and mannoprotein extraction optimization. After the selection of yeast strains and growth stage for subsequent extractions based on fluorescence intensity data, SB62 cells were subjected to various ultrasound and enzymatic conditions as single and combined treatments. Thereafter, the optimized conditions were additionally applied to SC01, MF77 and TD70. The obtained mannoprotein-rich extracts were preliminarily characterized and compared between yeast species and extraction conditions using protein and carbohydrate quantification and visualization methods.

### 3.1. Fluorescence Microscopy

**Figure 1:**
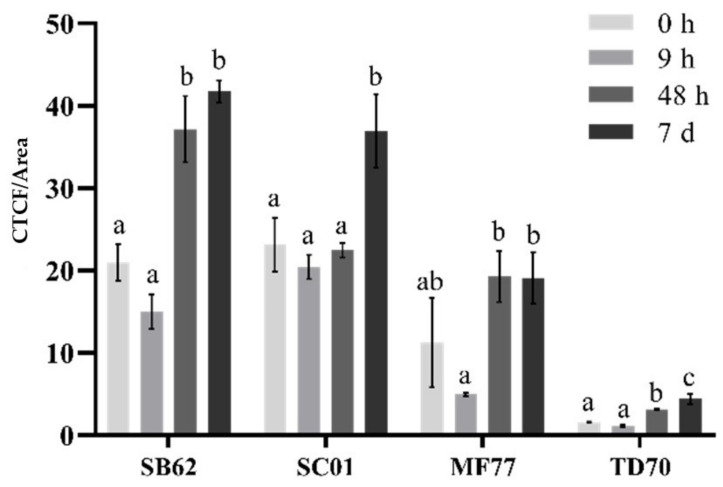
Corrected total cell fluorescence (CTCF) normalized by cell size, for samples of SB62, SC01, MF77 and TD70 collected at 0 h, 9 h, 48 h and 7 days (d) after inoculation into enrichment medium, and stained with FITC-ConA before fluorescence imaging. The data points shown are means for two biological staining repeats, calculated from the means of two technical repeats, derived from the means of five images, from which the mean CTCF/area for each cell captured was used. The error bars indicate standard deviation between biological repeats. Different letters indicate significant differences (*p* < 0.05) between samples collected at different times within each separate species as analyzed by one-way ANOVA and the Fisher’s LSD test.

**Figure A1:**
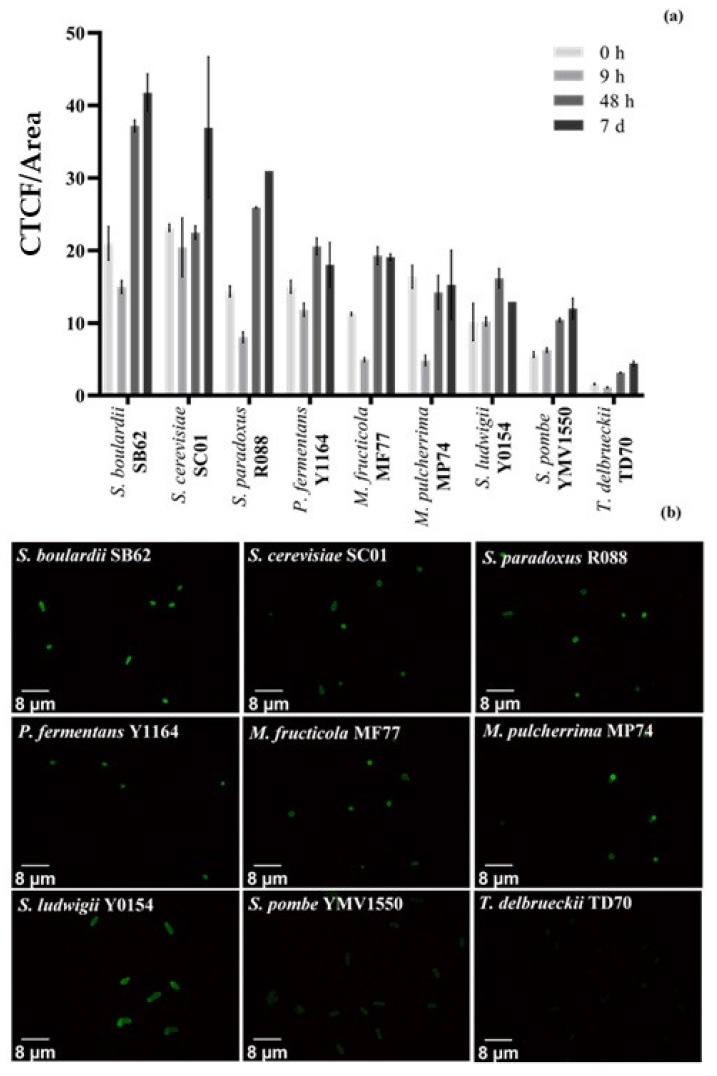
Fluorescent images and corrected total cell fluorescence (CTCF) obtained from the initial screening of all evaluated yeast strains: *S. boulardii* SB62, *S. cerevisiae* SC01, *S. paradoxus* R088, *P. fermentans* Y1164, *M. fructicola* MF77, *M. pulcherrima* MP74, *S. ludwigii* Y0154, *S. pombe* YMV1550 and *T. delbrueckii* TD70. (**a**) CTCF normalized by cell size, for samples collected at 0 h, 9 h, 48 h and 7 days after inoculation into enrichment medium and stained with FITC-ConA before fluorescence imaging. The data points shown are means for two biological staining repeats, calculated from the means of two technical repeats, derived from the means of five images, from which the mean CTCF/area for each cell captured was used. The error bars indicate standard deviation between biological repeats. (**b**) Fluorescent images taken of samples collected 48 h after inoculation into enrichment medium, using the Zeiss Axiocam 208 with constant exposure time. Scale bars represent 8 µm. Image analysis was performed using ImageJ software.

The fluorescence intensity of yeast cells grown to exponential (9 h), early stationary (48 h) or late stationary phase (7 days) in enrichment medium was examined microscopically, after staining with FITC-conjugated concanavalin A. Figure 1 depicts the corrected total cell fluorescence (CTCF) obtained from images taken from SC01, SB62, MF77 and TD70, the strains selected for further downstream evaluation due to the range of fluorescence represented. Fluorescence data and images from all 11 evaluated strains can be found in Appendix A (Figure A1). As depicted in Figure 1, after an initial insignificant decrease of fluorescence intensity from 0 h to 9 h after inoculation, a trend of significant increasing intensity was observed for all species from 9 h to 7 days after inoculation. For SB62, MF77 and TD70 the significant increase occurred between 9 h and 48 h, whereas this change was observed between 48 h and 7 days for SC01. Fluorescence intensities furthermore differed between yeast species at similar growth phases. Whereas intensities for SB62 and SC01were similar at all time-points with the exception of 48 h, MF77 fluorescence was notably lower, followed by that of TD70.

### 3.2. Mannoprotein Extraction Optimisation

#### 3.2.1. Variation of Ultrasound Treatment Parameters on Samples of SB62

**Figure 2:**
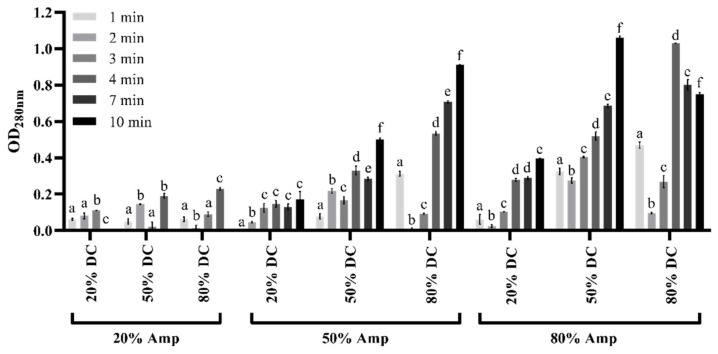
The optical density at 280 nm of supernatant samples collected after ultrasound treatment of SB62 cell suspensions. Sonication variables included percentage duty cycle (DC), percentage of the maximum amplitude (Amp) that was applied, and the total duration of ultrasound treatment in minutes. The data points for ultrasound durations of 7 and 10 min for 20% Amp are not shown in the figure due to technical problems with the sonication instrument associated with the cessation of sonication at this low intensity for prolonged periods of time. The data points shown are means for duplicate experiments and the error bars indicate standard deviation between replicates. Different letters indicate significant differences (*p* < 0.05) between sonication durations within each separate treatment as analyzed by one-way ANOVA and the Fisher’s LSD test.

Ultrasound as sole treatment was initially applied to cells of SB62, in order to determine the sonication parameters with effective mannoprotein release. Sonication duty cycle, intensity, and total duration were varied, and the optical density of the supernatant was measured at 280 nm as an indication of protein release. This measurement was employed as a rapid screening method in selecting effective parameters for subsequent applications. All of the varied parameters influenced the optical density values, the extent of which often depended on each other (Figure 2). With an increase in sonication amplitude, and therefore the intensity, also came an increase in optical density for all sonication lengths and duty cycles with the exception of the treatments at 7 and 10 min at 80% DC with 80% Amp. Similarly, an increase in duty cycle showed a trend of increasing OD_280nm_, except at 20% Amp and for the 10 min treatment at 80% DC with 80% Amp. Lastly, the effect of total sonication duration was typically an increase in optical density observed especially from 50% DC and 50% Amp and higher. However, at 80% DC and 80% Amp, the sonication treatments longer than 4 min yielded lower OD_280nm_ values than the shorter treatments. It should be noted that these treatments resulted in foaming of the samples which could significantly impact the intensity of sonication delivered.

#### 3.2.2. Variation of Enzymatic Treatment Parameters on Samples of SB62

**Figure 3:**
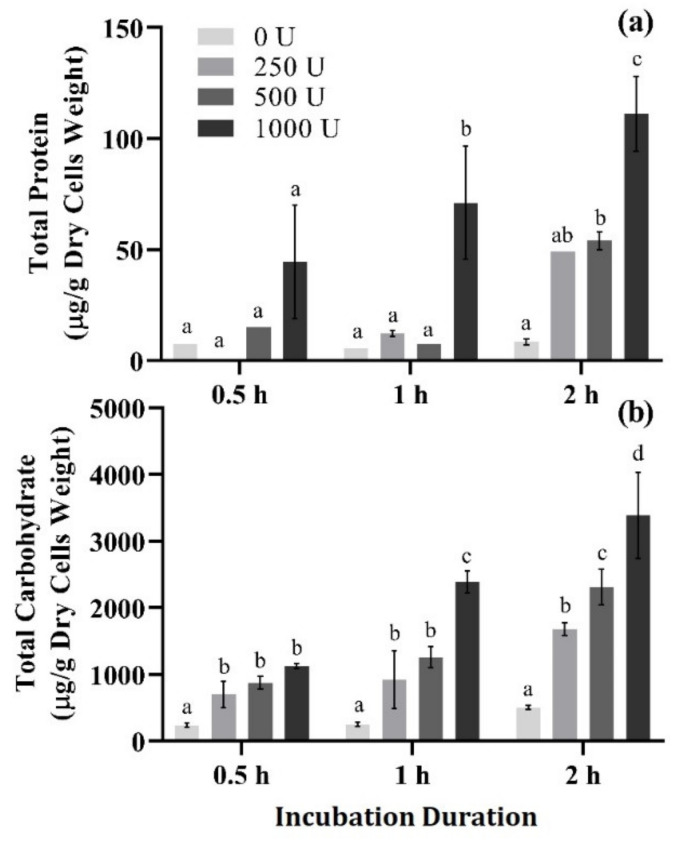
Total protein and carbohydrate content normalized by g dry weight cells of supernatant samples collected after enzymatic treatment of SB62 cell suspensions. Treatment variables included enzyme concentration (0, 250, 500 and 1000 U/g dry cells weight), and incubation duration (0.5, 1 and 2 h at 37 °C). (**a**) Total protein determined by BCA protein quantification. (**b**) Total carbohydrate determined by the phenol-sulphuric acid test. The data points shown are means for duplicate experiments and the error bars indicate standard deviation between replicates. Different letters indicate significant differences (*p* < 0.05) between samples treated with different enzyme concentrations within each separate incubation duration as analyzed by two-way ANOVA and the Fisher’s LSD test.

Enzymatic treatment of SB62 cells was performed with variations in enzyme concentration and incubation duration, and the total protein and carbohydrate was measured as an indication of mannoprotein release (Figure 3). With increasing enzyme concentration and incubation duration came an increase in both protein and carbohydrate concentration. The highest concentration yielded after the most concentrated treatment of 1000 U/g dry cells weight which were incubated for the longest duration of 2 h, did not exceed 111 μg protein and 3389 μg carbohydrate per g dry weight cells.

#### 3.2.3. Combination of Ultrasound and Enzymatic Treatment on Samples of SB62

**Figure 4:**
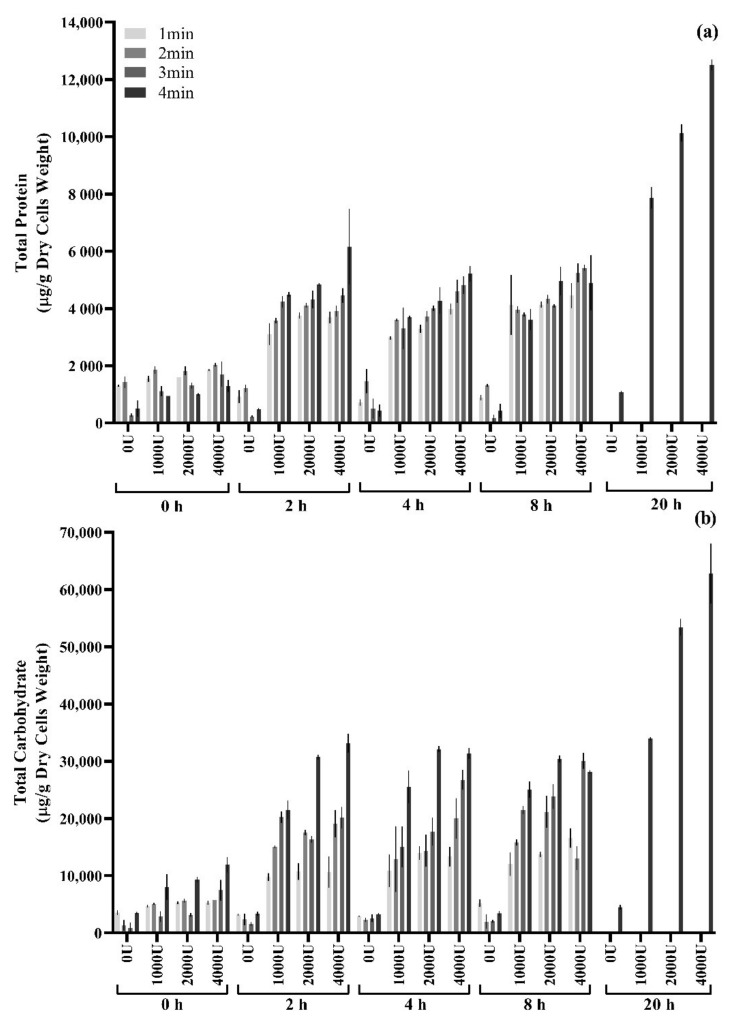
Total protein and carbohydrate content normalized by g dry cells weight of supernatant samples collected after enzymatic treatment (1000, 2000 and 4000 U lyticase/g dry cells weight incubated for 2, 4, 8 and 20 h at 37 °C, with negative controls excluding enzyme and/or incubation time) of SB62 cells collected after 1, 2, 3 and 4 min of ultrasound treatment (50% duty cycle and 80% amplitude), with negative controls of non-sonicated samples. (**a**) Total protein determined by BCA protein quantification. (**b**) Total carbohydrate determined by the phenol-sulphuric acid test. The data points shown are means for duplicate experiments and the error bars indicate standard deviation between replicates.

Samples of SB62 were subjected to ultrasound treatments performed at 50% duty cycle and 80% amplitude for total durations of 1, 2, 3 and 4 min, followed by enzymatic treatments at concentrations of 0, 1000, 2000 and 4000 U/g dry cells weight and incubation times of 2, 4, 8 and 20 h. The total protein and carbohydrate concentrations obtained are depicted in Figure 4. Increasing enzyme concentration and sonication duration had a small correlation with increasing protein and carbohydrate concentrations. However, extending the incubation duration to 20 h yielded the highest protein and carbohydrate concentrations. Indeed, whereas the range of protein and carbohydrate concentrations for samples sonicated for 4 min and incubated with 4000 U/g dry cells weight for 2 to 8 h remained 5000–6000 μg and 28,000–33,000 μg per g dry cells weight, respectively, samples incubated for 20 h yielded 13,000 μg protein and 63,000 μg carbohydrate per g dry cells weight under the same sonication duration and enzyme concentration.

#### 3.2.4. Ultrasound and Enzymatic Treatment of Additional Yeast Species

**Figure 5:**
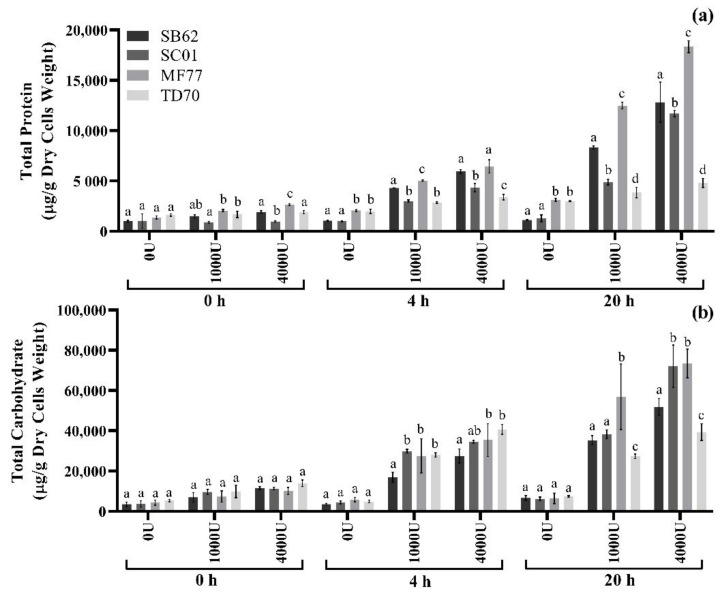
Total protein and carbohydrate content normalized by g dry cells weight of supernatant samples collected after enzymatic treatment (1000 and 4000 U lyticase/g dry cells weight incubated for 4 and 20 h at 37 °C, with negative controls excluding enzyme and/or incubation time) of SB62, SC01, MF77 and TD70 cells collected after 4 min of ultrasound treatment (50% duty cycle and 80% amplitude). (**a**) Total protein determined by BCA protein quantification. (**b**) Total carbohydrate determined by the phenol-sulphuric acid test. The data points shown are means for triplicate experiments and the error bars indicate standard deviation between replicates. Different letters indicate significant differences (*p* < 0.05) between species within each separate extraction condition as analyzed by two-way ANOVA and the Fisher’s LSD test.

Combined ultrasound and enzymatic treatments were then applied to samples of SB62, SC01, MF77 and TD70. Samples were sonicated for 4 min at 50% duty cycle and 80% amplitude and subjected to enzymatic digestion at 1000 and 4000 U lyticase per g dry cells weight for 4 and 20 h incubation duration. The total protein and carbohydrate concentrations obtained are depicted in Figure 5. The trend of increasing protein and carbohydrate extraction with increasing enzyme concentration and incubation duration was consistent between species, although the concentrations yielded at similar treatment conditions differ. MF77 and SB62 protein concentrations exceeded those of SC01 and TD70 for all treatments, with yields of 18,000 and 13,000 μg/g dry cells weight, respectively, in samples treated with 4000 U/g dry cells weight for 20 h. On the contrary, carbohydrate concentrations remained similar between species until 20 h of incubation, when SC01 and MF77 samples showed higher yields at 72,000 and 74,000 μg/g dry cells weight, respectively, in samples treated with 4000 U/g dry cells weight.

**Figure 6:**
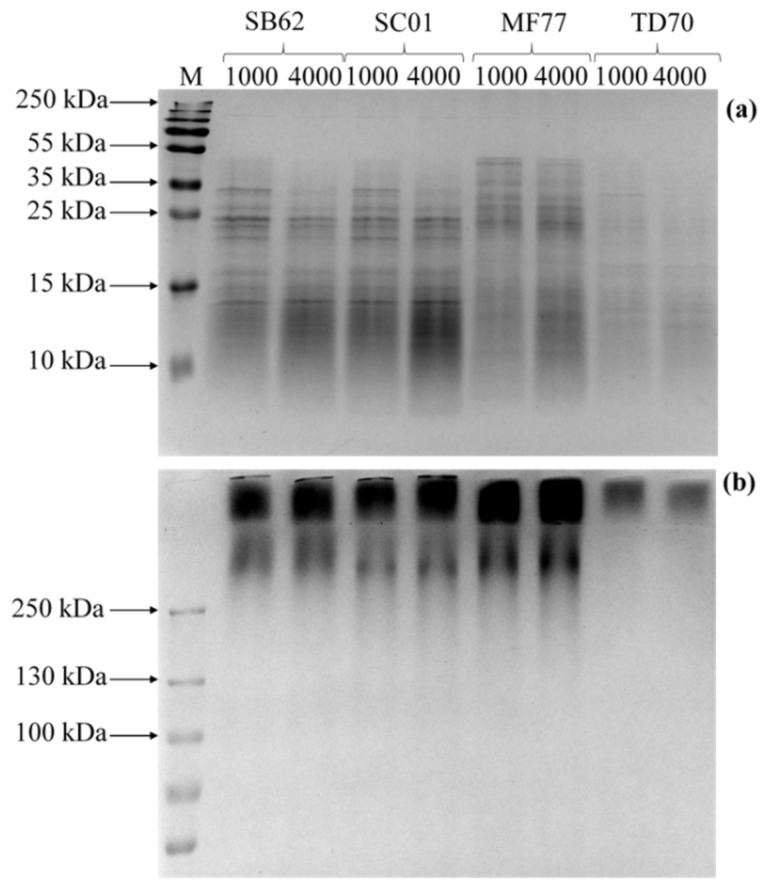
SDS-PAGE visualization of proteins and carbohydrates obtained from samples of SB62, SC01, MF77 and TD70 after ultrasound treatment and incubation with 1000 U or 4000 U lyticase per g dry cells weight, for 20 h. (**a**) Visualization of proteins after Coomassie staining. (**b**) Visualization of carbohydrates after PAS staining.

Electrophoretic visualization of proteins and carbohydrates obtained from samples of SB62, SC01, MF77 and TD70, after ultrasound treatment and incubation with 1000 U or 4000 U lyticase per g dry cells weight for 20 h, are depicted in Figure 6. Staining with Coomassie blue revealed protein bands smaller than 55 kDa in all samples. On the other hand, PAS staining revealed the presence of carbohydrates larger than 250 kDa, a proportion of which did not enter the resolving gel during the period of electrophoresis.

**Figure 7:**
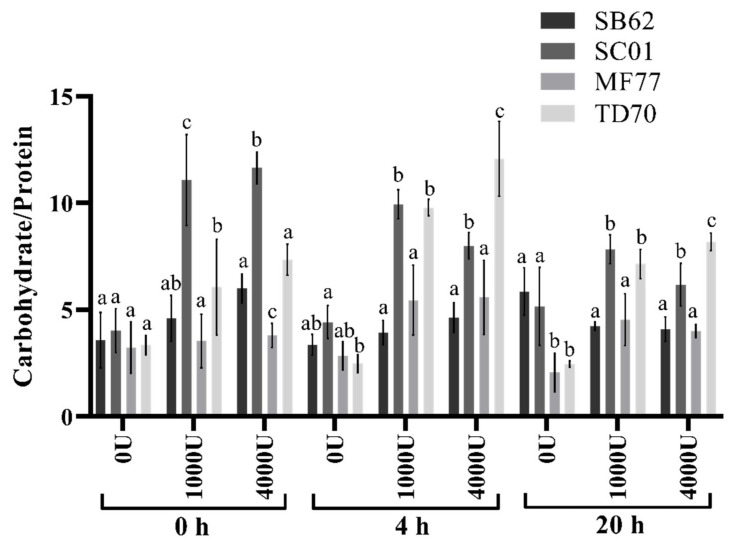
Carbohydrate to protein ratios in samples collected after enzymatic treatment (1000 and 4000 U lyticase/g dry cells weight incubated for 4 and 20 h at 37 °C, with negative controls excluding enzyme and/or incubation time) of SB62, SC01, MF77 and TD70 cells collected after 4 min of ultrasound treatment (50% duty cycle and 80% amplitude). Different letters indicate significant differences (*p* < 0.05) between different species within each separate extraction condition as analysed by two-way ANOVA and the Fisher’s LSD test.

The ratios of carbohydrate to protein concentrations displayed in Figure 5 are represented in Figure 7. The proportions of carbohydrate to protein in the samples obtained after ultrasound and enzymatic treatment did not seem much affected by an increase in enzyme concentration from 1000 U to 4000 U/g dry cells weight, however the carbohydrate/protein ratio did show a decrease with increasing incubation time from 4 h to 20 h. Furthermore, differences in the proportion of carbohydrate to protein were evident between species, with TD70 and SC01 showing higher carbohydrate/protein ratios for almost all treatments, in comparison to MF77 and SB62.

## 4. Discussion

In this study, fluorescence microscopy analysis of concanavalin A-bound yeast cells and the preliminary evaluation of mannoprotein extracts using colorimetric assays were employed to investigate the cell wall mannoprotein content and composition of four different yeast species. Previous studies have demonstrated the extent of mannoprotein release by various *Saccharomyces* and non-*Saccharomyces* strains into the medium, such as in wine during fermentation or aging on yeast lees, but information regarding the in-situ levels of mannoprotein production is limited [28,30,31,32,43,44].

Concanavalin A (ConA) is a lectin of the jack bean (*Canavalia ensiformis)* that binds specifically to the α-ᴅ-glucopyranosyl, α-ᴅ-mannopyranosyl, β-ᴅ-fructofuranosyl, or α-ᴅ-arabinofuranosyl residues of polysaccharides [45]. Due to its comparably higher affinity to α-linked mannose homopolymers, it has been used extensively as a tool to study mannose-rich glycans such as those found in the yeast cell wall [46,47,48,49,50,51]. In this study, ConA was used as a marker for the evaluation of the relative abundance of mannoprotein in the cell wall of different yeast species at various points of growth, as a preliminary investigation into the impact of species and growth phase on mannoprotein content.

Indeed, there is evidence for changes in yeast cell wall composition dynamics as growth progresses from exponential to stationary phase, as further suggested by the increasing fluorescence over time of the yeasts investigated in this study [20,22,46]. While certain cell wall proteins related to bud formation and cell separation are upregulated during the cell cycle of exponential growth in *S. cerevisiae*, during stationary phase the cell wall significantly thickens which may largely be due to the enhanced expression of heavily *N*-glycosylated cell wall proteins such as Sed1p [52]. With this in mind, the initially high fluorescence values at 0 h can possibly be explained by the growth phase of the inoculated cells, having been aliquoted from overnight cultures approaching stationary phase (see Figure 1).

The differences in fluorescence between yeast species furthermore suggests different levels of cell wall mannoprotein production and/or mannosylation. It is possible that the mannoprotein content of the cell wall differed between these yeasts, perhaps due to species- and/or strain-dependent responses to external stimuli. Indeed, it is evident that environmental conditions and stress factors have a direct impact on the continuous changes and dynamics of *S. cerevisiae’s* cell wall architecture and mannoprotein-encoding genes [22,37,52,53]. While *S. cerevisiae* is most studied regarding cell wall responses, other yeast species may exhibit different stress-induced phenotypes [43]. However, it is also possible that the nature of ConA binding resulted in a biased representation of fluorescence towards cells harboring mannoproteins with greater affinity to the FITC-conjugated lectin [45]. For instance, it has been shown that the mannose/glucose ratio of mannoproteins may vary between species and strains, and as ConA shows greater affinity towards mannose, the results may be skewed to suggest that cells with higher concentrations of mannose residues also carry more total mannoprotein [28,32]. It is furthermore possible that the mannoprotein’s degree of glycosylation, and the extent of polysaccharide polymerisation (both of which may differ between species and strains) had an impact on ConA binding. Lastly, mannoprotein density on the cell wall itself, as well as cell wall surface area variations between species with different cell sizes, could also contribute to levels of ConA binding on a basis other than mannoprotein concentration [46]. Nevertheless, as depicted in Figure 1, the fluorescence intensity data obtained in this study does show a trend consistent across the evaluated strains that strongly suggests a species and growth effect on mannoprotein content.

Prior to mannoprotein extraction from all species under investigation in this study, *S. boulardii* SB62 was subjected to various ultrasound and enzymatic treatments. Due to its relatively higher levels of fluorescence, suggestive of higher mannoprotein levels, this strain was selected for optimization purposes before testing the selected parameters on the other species. The growth phase for sampling and subsequent extractions was selected based on the fluorescence intensity results. The maximum fluorescence for all species was obtained in early stationary phase, with only *S. cerevisiae* SC01 showing a significantly increased fluorescence at late stationary phase as compared to early stationary. It was therefore decided to perform extractions at early stationary phase in order to optimize mannoprotein concentrations while ensuring the effectiveness of enzymatic digestion, which has previously been shown to be hindered with the thickening of the cell wall as stationary phase progresses [54].

Various sonication and enzymatic treatments were applied to SB62 cells separately, before selected parameters from each were used in combination. Sonication variables investigated in this study included the total duration of sonication, the duty cycle of sonication (percentage of time during which sonication was occurring) and intensity of sonication delivered to the samples (percentage of the maximum possible amplitude deliverable by the instrument), all of which have demonstrated significant influence on the efficiency of sonication [55,56,57,58]. Optical density measurements at 280 nm were used as an indication of protein release due to the effectiveness of ultrasound treatments, as has been performed previously [55]. On this basis, treatments of 4 min and longer with a duty cycle of 80% at 50% of the maximum possible amplitude, or a duty cycle of 50% or 80% at 80% of the maximum possible amplitude, yielded the highest protein concentrations (see Figure 2). However, treatments exceeding 4 min frequently led to foaming of the sample, whereas increased sample temperatures were observed for treatments longer than 4 min and when performed with 80% duty cycle. Both of these phenomena have been shown to affect sonication efficiency, and in the case of temperature, even influence the protein/polysaccharide ratio in the obtained sample [57]. It was therefore decided to apply the ultrasound treatment parameters of 50% duty cycle and 80% of the maximum possible amplitude to future samples, for total sonication durations not exceeding 4 min. When subjected to enzymatic treatment alone, it became clear that protein and carbohydrate concentrations increased with an increase in both enzyme concentration and the duration of incubation for the parameters tested (see Figure 3). However, the yield obtained at 0.35% of the total cell dry weight was much lower than expected. The expected yield of mannoprotein ranges between 7% and 12% of the total cell dry weight [59]. It was therefore decided to increase both the enzyme concentration, including treatments of 2000 and 4000 U/g dry cells weight in addition to the previously used 1000 U/g dry cells weight, and incubation duration, and combine with sonication in an attempt to improve mannoprotein yield.

It became clear that a combination of sonication and enzymatic digestion resulted in greater yields of protein and carbohydrate than when either method was applied alone. Sonication of SB62 cells for 4 min followed by enzymatic treatment with 4000 U/g dry cells weight for 20 h obtained a combined protein and carbohydrate yield of 7.6% of the total cell dry weight (see Figure 4). This could be due to the cumulative effect of the combined treatments such that differently bound mannoproteins were released by the action of the different methods. Physical and chemical methods have shown effective release of non-covalently bound mannoproteins that are loosely associated with the cell, whereas β-glucanase is necessary for the release of covalently bound mannoprotein [4]. It is also possible that the damage to the cell walls due to the shear forces generated by sonication increased accessibility for enzymatic activity, thus promoting further mannoprotein release. Nevertheless, the concentrations presented in this study cannot be attributed solely to the composition of mannoproteins, as this is necessarily not the only source of proteins and carbohydrates contained in the extract obtained using the ultrasound and enzymatic methods employed here. Indeed, components such as intracellular proteins and degraded cell wall β-glucans would also contribute to the total protein and carbohydrate concentrations obtained. The ratios of protein/carbohydrate discussed thus describe the total extract, including but not only of that contained by the mannoprotein.

When the combined sonication and enzymatic treatments were applied to additional yeast species, a consistent trend of increasing protein and carbohydrate concentrations measured by colorimetric assays were observed with an increase in enzyme concentration and incubation duration (see Figure 5). The carbohydrates obtained after 20 h incubation with 1000 and 4000 U/g dry cells weight are also reflected in the PAS-stained electrophoresis gels (Figure 6b). This provides additional evidence for the presence of carbohydrates. Mannoproteins and glucans are the major cell-wall carbohydrates, but given the nature of the analytical technique used, bands in Figure 6b most likely correspond to yeast mannoproteins, as their protein domain allows for their ability to enter at least partially the SDS-PAGE gels [17,60,61]. Additionally, the β-glucanase treatment aims at releasing fractions rich in mannoproteins, although possibly still bearing some small fragments of the glucan to which it was covalently bound in the yeast cell wall. Thus, these results strongly suggest the presence of mannoproteins. However, the protein moiety of the mannoprotein could not be visualized after staining with Coomassie blue. This could be due to an insufficient concentration of mannoprotein in the sample loaded, the low sensitivity of Coomassie blue, or steric hindrance of the carbohydrate on the highly glycosylated mannoprotein leading to failure of detection [62]. Furthermore, it is important to note the substantial intracellular protein content also extracted during the ultrasound and enzymatic treatments as visualized on the Coomassie-stained gel (see Figure 6), confirming that the protein quantified by colorimetric assays cannot be solely attributed to mannoprotein. Nevertheless, although the ratio of carbohydrate to protein of the extracted mannoproteins is thus likely higher than shown for the total extract, the trend of compositional differences between species and extractions should still be considered.

Most investigations aimed at characterizing non-*Saccharomyces*-derived mannoproteins have focused on the compounds released from the cells into the surrounding medium during alcoholic fermentation or aging on the lees [28,30,31,32,43,44,63]. It is thus frequently unclear whether the levels of polysaccharide measured are due to the levels of mannoprotein in the yeast cell wall and their in-situ composition, or to the species-specific release of mannoproteins as influenced by environmental conditions. Nevertheless, trends of differing mannoprotein composition between various yeast species in terms of carbohydrate and protein content such as those described here have similarly been observed in these studies. For instance, in comparison to other *Saccharomyces* and non-*Saccharomyces* yeast strains, mannoproteins released by *T. delbrueckii* have previously shown increased ratios of carbohydrate to protein, an observation similar to that found in this study when strains were incubated with 4000 U lyticase/g dry cells weight [28]. However, extraction conditions also played a role in the ratio of carbohydrate to protein observed. As the duration of time that cells were incubated with enzyme increased, the amount of carbohydrate decreased relative to that of released protein, which could be due to the increasing extraction of intracellular proteins over time (see Figure 7).

The optimization of mannoprotein extraction is an important step towards the preparation of well-defined fractions that may ultimately be subjected to in-depth characterization and application to the food and beverage industry, and an understanding of the impact that different extraction conditions have on the obtained sample is crucial. Insights into how mannoproteins from different origins impact aspects of wine quality would contribute towards a framework for the selection of strains and extraction methods for obtaining exogenous mannoprotein preparations that achieve the desired oenological outcome. Such information would furthermore expand our understanding regarding the cell wall structural diversity that exists between yeast species.

## 5. Conclusions

In this study, evaluation of the combined use of ultrasound and enzymatic treatment on cells from different yeast species revealed that ultrasound treatment followed by 20 h incubation with β-glucanase yielded the highest concentrations of carbohydrate and protein. Preliminary investigations into the composition of the mannoprotein-rich extracts obtained suggest an impact of incubation duration on carbohydrate/protein ratio, which is an important factor to consider for applications such as wine protein haze reduction and tartrate stabilization. However, this ratio cannot be attributed solely to the composition of the mannoprotein contained in the extract, but necessarily also reflects the presence of components such as intracellular proteins and fragments of β-glucans released due to the ultrasound and enzymatic methods employed in this study. Therefore future studies should include targeted and more in-depth characterization to confirm that these findings can be attributed to the mannoprotein itself, upon isolation from the extract. Should species-specific differences in carbohydrate content subsequently be confirmed, strain selection may also be critical for obtaining mannoproteins of interest to specific applications. Nevertheless, further research is necessary for characterization of the mannoproteins obtained in order to relate their structural features and differences to their potential impact on wine properties.

## Data Availability

Not applicable.
